# The human mitochondrial transcription factor A is a versatile G-quadruplex binding protein

**DOI:** 10.1038/srep43992

**Published:** 2017-03-09

**Authors:** Sébastien Lyonnais, Aleix Tarrés-Soler, Anna Rubio-Cosials, Anna Cuppari, Reicy Brito, Joaquim Jaumot, Raimundo Gargallo, Marta Vilaseca, Cristina Silva, Anton Granzhan, Marie-Paule Teulade-Fichou, Ramon Eritja, Maria Solà

**Affiliations:** 1 Structural MitoLab, Structural Biology Unit, Molecular Biology Institute of Barcelona (CSIC), Barcelona, 08028, Spain; 2 Department of Chemical Engineering and Analytical Chemistry, University of Barcelona, Barcelona, 08028, Spain; 3 Mass Spectrometry Core Facility, Institute for Research in Biomedicine, IRB Barcelona, 08028 Barcelona, Spain; 4 CNRS UMR9187, INSERM U1196, Institut Curie, Université Paris-Sud, 91405 Orsay, France; 5 IQAC-CSIC, CIBER-BBN, Barcelona, E-08034, Spain

## Abstract

The ability of the guanine-rich strand of the human mitochondrial DNA (mtDNA) to form G-quadruplex structures (G4s) has been recently highlighted, suggesting potential functions in mtDNA replication initiation and mtDNA stability. G4 structures in mtDNA raise the question of their recognition by factors associated with the mitochondrial nucleoid. The mitochondrial transcription factor A (TFAM), a high-mobility group (HMG)-box protein, is the major binding protein of human mtDNA and plays a critical role in its expression and maintenance. HMG-box proteins are pleiotropic sensors of DNA structural alterations. Thus, we investigated and uncovered a surprising ability of TFAM to bind to DNA or RNA G4 with great versatility, showing an affinity similar than to double-stranded DNA. The recognition of G4s by endogenous TFAM was detected in mitochondrial extracts by pull-down experiments using a G4-DNA from the mtDNA conserved sequence block II (CSBII). Biochemical characterization shows that TFAM binding to G4 depends on both the G-quartets core and flanking single-stranded overhangs. Additionally, it shows a structure-specific binding mode that differs from B-DNA, including G4-dependent TFAM multimerization. These TFAM-G4 interactions suggest functional recognition of G4s in the mitochondria.

Under physiological conditions, RNA or DNA strands containing tracts of guanines can fold into structures called G-quadruplexes (G4). The main unit of a G4 consists of G-quartets formed by four guanine residues arranged in a planar ring through Hoogsteen hydrogen bonding. The stacking of two or more G-quartets and the coordination of cations between them generate a G4, which can assemble from one (intramolecular), two (bimolecular) or four (tetramolecular) strands^1^. The relative orientation of the guanine tracts, the nucleotide sequences and length between them define parallel or antiparallel G4 topologies. Sequences prone to form G4 structures are widely present in the genomes of all organisms and in a non-random distribution that correlates with functionally important chromosomal regions^2^. Structure-specific antibodies and synthetic G4 ligands were used to visualize these structures in human cells and to identify G4 sequences from human genomic DNA^3^. Their *in vivo* functions involve transcription regulation, DNA replication and genomic stability, translation and RNA maturation, telomere biology and replication origin positioning (reviewed in refs 1,4, 5, 6, 7). In addition, G4s have been identified as regulatory targets of transcriptional, translational and epigenetic cellular factors^5^ and numerous reports have indirectly demonstrated their involvement in human diseases^8^. G4-specific recognition by binding proteins depends on structural features such as the groove of the G-quartet barrel, the terminal G-quartets, the duplex region, the DNA duplex/G4 junction or a combination of these elements^9^,^10^,^11^,^12^,^13^.

The presence of G4s in human mitochondrial DNA (mtDNA) has been recently addressed. The mtDNA is a small circular genome encoding 13 respiratory-chain subunits, packaged and organized into nucleoprotein complexes (nucleoids) in the mitochondrial matrix. The nucleoids contain transcription and replication machineries imported from the cytoplasm and their activities are tightly regulated. Human mtDNA contains a cytosine-rich light (L) strand and a complementary heavy (H) strand. The latter is highly enriched in a series of repeated guanines^14^ prone to form G4^15^,^16^,^17^ that have been recently visualized in cancer cells^18^. mtDNA contains a control region which contains the light and heavy strand transcription promoters (LSP and HSP, respectively), the H-strand origin of replication (O_H_) and three conserved sequence blocks (CSBI-III, downstream to LSP). A stable R-loop forms downstream to LSP as a result of premature transcription termination at CSBII. This premature termination has been related to the formation of a G4 at CSBII that aborts transcription. This additionally generates a 3′ end for H-strand replication priming, suggesting a G4-mediated transcription/replication switch^19^,^20^,^21^,^22^. Computational analyses have correlated mtDNA deletions with non-B DNA structures^23^ and G4 prone-motifs appear to be preferentially located near deletion break-points observed in patients with genetic disorders^16^,^17^. Thus, it is likely that mtDNA forms G4 structures as in nuclear DNA, either endowed with a function or potentially causing mtDNA instability and, possibly, are recognized by mitochondrial proteins. LON, the major protease of the mitochondrial matrix, was found to bind mtDNA and specifically to a G-rich sequence at LSP that forms a tetramolecular G4 *in vitro*^24^. The mitochondrial GTPase NOA1 was also found to bind specifically to oligonucleotides that fold into G4s^25^.

The mitochondrial Transcription Factor A (TFAM) is a key component of mtDNA transcription and is the main factor for nucleoid compaction and mtDNA maintenance^26^,^27^,^28^,^29^. TFAM is a member of the High-Mobility Group (HMG)-box protein superfamily. HMG-box proteins are abundant, essential, ubiquitously expressed. They are non-histone architectural elements that modify the structure of the DNA and facilitate DNA transcription, replication and recombination^28^,^30^. TFAM, which contains two HMG-box domains, coats and condenses the entire mtDNA by non-specific binding and multimerization^26^,^31^,^32^. On the other hand, TFAM activates transcription by binding and bending specific sequences at HSP or LSP^29^. Like other HMG-box proteins, TFAM also recognizes tRNA and distorted structures, such as DNA/RNA-containing 4-way junctions^33^,^34^, base bulges, cis-platinum adducts or damaged DNA^35^,^36^. In the mitochondrial matrix TFAM levels are regulated by LON^37^. *In vitro*, TFAM proteolysis is strongly inhibited by fragments of the LSP or by the G4-forming DNA recognized by LON, suggesting a DNA-dependent cleavage of TFAM^37^. The G4-binding GTPase NOA1 co-purifies with TFAM^38^, and TFAM is immunoprecipitated by the mitochondrial member of the RECQ family of G4-specific helicases, RECQL4^39^. All these clues pointed out TFAM as a candidate in the pool of mitochondrial proteins that recognize G4s. Guided by this hypothesis, we found and report here that, indeed, human TFAM binds with high affinity and great versatility to DNA and RNA G4s in a structure-specific manner. These results and the evidences of G4 formation in mtDNA suggest that TFAM binding to these structures might be tightly regulated in the mitochondria.

## Results

### CSBII-G4 DNA binds human TFAM from mitochondria extracts from HeLa cells

**Figure 1:**
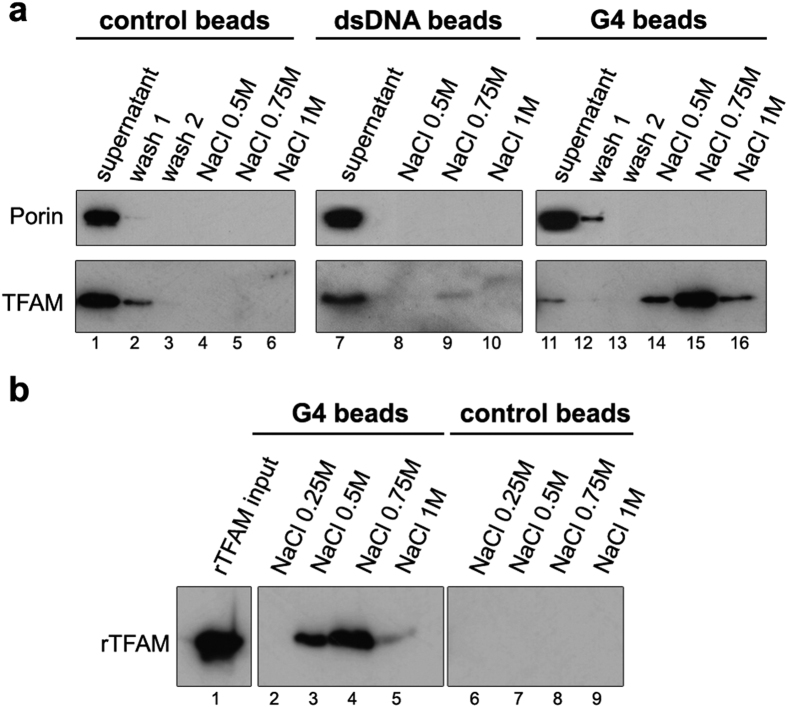
TFAM is captured from mitochondrial extracts with CSBII G4-DNA by pull-down assays. Experiments were carried out with bulk magnetic beads (control beads), beads coated with CSBII DNA-G4 (G4 beads) or with the corresponding CSBII dsDNA fragment (dsDNA beads). Bound proteins were revealed by Western blot after SDS-PAGE. (**a**) Pull-down assays using mitochondrial fractions incubated with the indicated beads in the presence of 0.5% Triton X-100. Lane ‘Supernatant’ corresponds to the mitochondrial extract after incubation with each type of beads, and which was used as a control of the input material with Porin as a reference. The NaCl concentration at each elution step is indicated. For the naked and G4 beads, the gels show two washing steps to evince the specificity of the assay. The amounts of TFAM at the supernatants are inversely proportional to the ones eluted with NaCl. (**b**) Pull-down assays performed with recombinant TFAM (rTFAM) incubated with G4 (lanes 2–5) or control beads (6–9) that show non-specific binding upon blockage with appropriate buffer. Bound rTFAM was eluted with the indicated amounts of NaCl after three washings steps. Note the efficient blockage of TFAM binding to the control beads.

In order to isolate potential G4-DNA binding proteins from mitochondria, we carried out pull-down assays similar to previous experiments performed for cytoplasmic G4-RNA binding proteins^40^. Streptavidin-coated magnetic beads were functionalized with biotinylated 30mer oligonucleotides spanning the human CSBII H-Strand sequence folded into G4s (Table 1 and Supplementary Fig. S1)^15^,^20^,^22^ or the corresponding double-stranded (ds) CSBII fragment. The G4-coated beads were incubated with mitochondria-enriched fractions from HeLa cells, in presence of competitor DNA and 0.5% Triton X-100, which smoothly disrupts mitochondrial membranes while keeping intact the matrix proteins and DNA components^41^. The G4-bound proteins were next released from the beads by an increasing salt gradient and separated by SDS-PAGE. Western blot analysis revealed the capture of mitochondrial TFAM, which was eluted at 0.5–0.75 M NaCl (Fig. 1). The high ionic strength indicated a strong and specific binding to the G4 bait. Bulk magnetic beads or dsDNA-coated beads showed undetectable or very low TFAM binding in the same conditions (Fig. 1a). A similar experiment with recombinant TFAM (Fig. 1b) also showed strong binding to G4-coated beads, while binding to DNA-free beads was again undetectable. These first results demonstrated that endogenous TFAM from mitochondrial extracts strongly binds exogenous G4 DNA, which can compete the mtDNA binding. This prompted us to characterize more precisely the recognition of G4 by recombinant TFAM *in vitro*.

### Human TFAM is a versatile G4-binding protein *in vitro*

**Figure 2:**
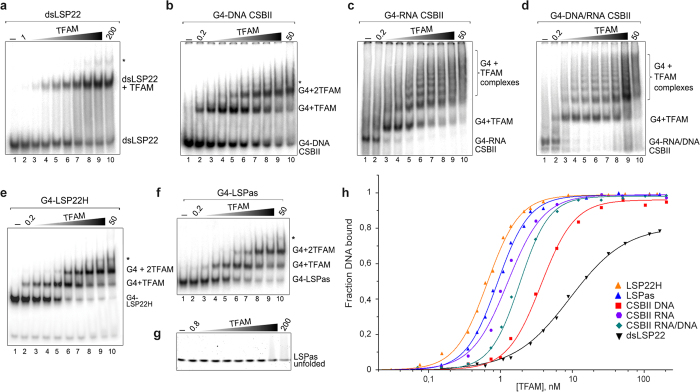
TFAM recognizes DNA and RNA oligonucleotides folded into G4 structures. (**a–g)** EMSA of the indicated ^32^P-labelled substrates (0.5 nM) incubated with increasing concentrations of rTFAM (first and last dilutions, in nM, are indicated for each gel in lane 2 and 10, respectively). In (**g**), the unfolded LSPas was obtained by alkaline denaturation and neutralization of the G4 before immediate incubation with TFAM. (**h**) Experimental points from the mobility-shift titrations exemplified in panels a-g for binding quantification and curve fitting using the Hill equation. The fraction of the total DNA bound was used for the G4 substrates. The solid curves result from fitting the data according to Eq. (1). Titration for dsLSP22 was not carried beyond 80% of saturation, because TFAM aggregation occurs at high protein concentration (e.g. >300 nM).

TFAM binding to a small library of G4s was followed by Electrophoretic Mobility Shift Assay (EMSA). We generated G4-DNA, RNA and DNA/RNA structures with oligonucleotides from the CSBII sequence (Fig. S1). The substrates were 5′-end labelled with ^32^P, incubated in presence of increasing amounts of TFAM and analysed by native PAGE. Binding was compared to a control 22 bp dsDNA fragment containing the TFAM binding site at LSP (dsLSP22), which is the best-characterised TFAM binding sequence. As reported previously^42^, TFAM efficiently recognized this dsDNA fragment and formed a single-band up-shift corresponding to a TFAM/dsDNA complex (Fig. 2a). The highest TFAM concentrations produced minor high molecular weight (MW) species (* in lanes 8–10) likely due to oligomerization^32^,^42^. In contrast, increasing TFAM concentration in presence of CSBII-DNA G4s showed formation of a TFAM/G4 complex that converted into a second shift after half-saturation of the DNA (Fig. 2b). TFAM was thus capable of recognizing G4 DNA, confirming its affinity for the aforementioned G4 bait. As with dsLSP22, the experiment revealed minor bands of higher MW species with an excess of TFAM, which migrated as smearing species resembling protein:DNA aggregates or high MW multimers. The presence of RNA in G4s resulted in a different type of binding: CSBII-RNA and CSBII-DNA/RNA G4s incubated with TFAM revealed a surprising band-shift pattern of a TFAM/G4 complex that converted, after half-saturation, into multiple regularly-spaced bands of lower mobility (Fig. 2c,d, lanes 3–10). As with the other substrates, very high MW protein/DNA complexes and smearing species were observed with an excess of TFAM. This multimerization and protein/DNA aggregation was reproduced by incubating TFAM with a 22bp-long CSBII-derived RNA/DNA hybrid G4 construct^22^ (Fig. S3d). Control constructs with the G4 structures substituted by single-stranded (ss-) overhangs yielded a single binding event, as seen for LSP22 dsDNA (Fig. S3b,c). To our knowledge, the multiple-band pattern has not been described for TFAM and suggests important complex oligomerization and/or an alternative binding mechanism, which could be induced by the G4 topology and/or its RNA content.

We next tested TFAM binding to the LON binding sequence LSPas d(AATAATGTGTTAGTTGGGGGGTG)^24^, which protects TFAM from degradation by LON *in vitro*^37^. This sequence forms highly stable tetramolecular G4s with six stacked G-quartets^24^. Since it overlaps the H-strand sequence of the TFAM binding site at LSP d(GTTAGTTGGGGGGTGACTGTTA) (LSP22H), we also produced G4s from LSP22H, which folded into very stable parallel tetramolecular species (Figs S1 and S2). The EMSA showed that both tetramolecular G4s were strongly bound by TFAM in a well-defined two-step mechanism (Fig. 2e,f), as observed for CSBII G4-DNA. As reported previously^33^, TFAM did not bind unfolded ssDNA, or traces of LSP22H oligonucleotide (Fig. 2e), or pre-denatured LSPas (Fig. 2g).

To evaluate TFAM binding to intra-molecular G4s, we chose two well-characterized G4-forming sequences from promoter regions in human nuclear DNA: the c-myc promoter sequence^43^ and the insulin-linked polymorphic region (ILPR) of the insulin gene promoter^44^. Intramolecular G4s folded in presence of potassium were confirmed by CD, which showed a typical spectrum of parallel structures for c-myc^43^, while a majority of antiparallel structures was found for ILPR (Fig. S4a)^45^. Both G4s were recognized by TFAM, producing a single up-shifted complex with c-myc and a double-shifted band pattern with ILPR (Fig. S4b). Formation of protein/G4 complexes concurred with a background of smearing species suggesting some dissociation during electrophoresis or important structural heterogeneity. We determined the apparent Kd for each substrate by quantifying bound and unbound species from the EMSA (Table 1). For comparison, binding data were fitted using the Hill equation (Eq. 1) by summing all shifted species. Binding to dsLSP22 yielded an apparent Kd of 9.2 nM, in agreement with the 5–10 nM range measured by others^28^,^33^,^42^. Under the same experimental conditions, apparent Kd in the 1–4 nM range was found for CSBII G4-DNA (Kd_DNA_ = 3.65 nM), G4-RNA (Kd_RNA_ = 1.25 nM) and G4-DNA/RNA (Kd_RNA/DNA_ = 1.85 nM). Intramolecular nuclear G4s showed lower affinity (Kd_c-myc_ = 53 nM and Kd_ILPR_ = 118 nM). Regarding tetramolecular G4s, the interaction was of very high affinity (Kd_LSP22H_ = 0.65 nM; Kd_LSPas_ = 0.89 nM). Notably, a 10–12-fold lower TFAM concentration was needed to bind 50% of total G4-LSP22H compared to that needed to bind 50% dsLSP, the key target sequence of TFAM on mtDNA.

**Figure 3:**
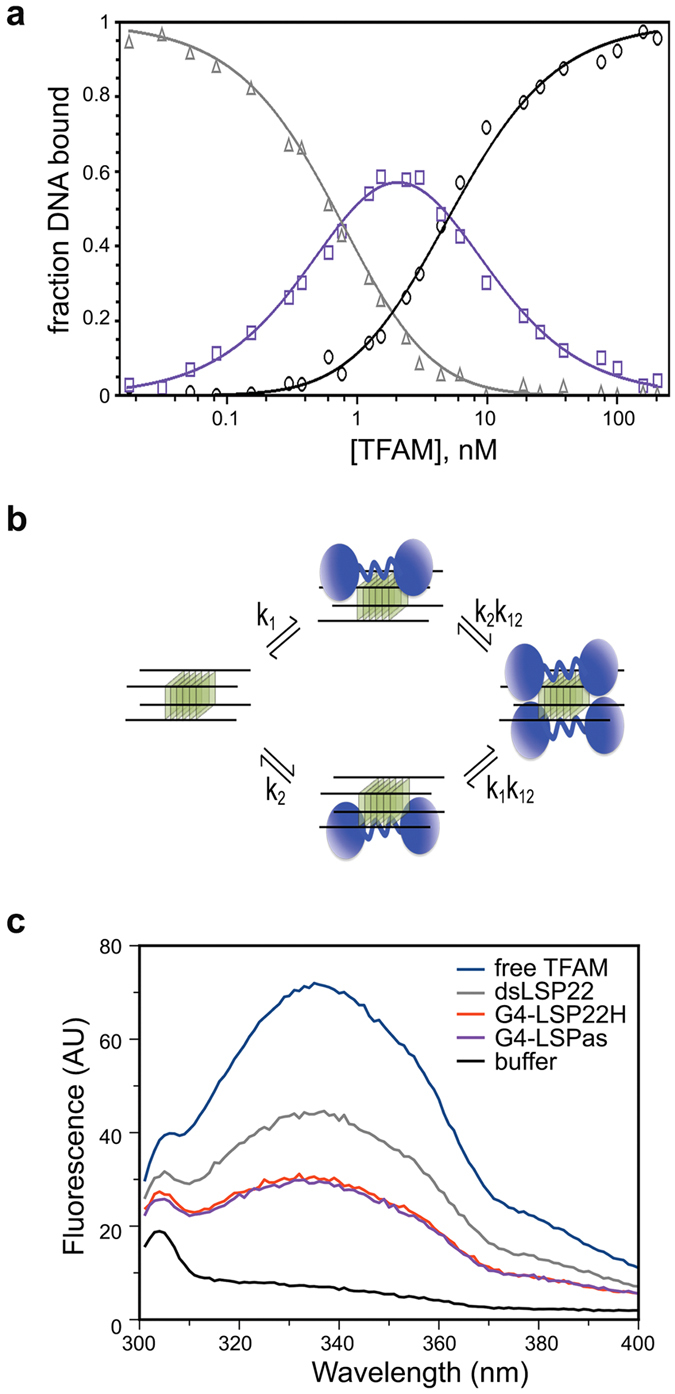
Two TFAM can bind a single tetramolecular G4, which is not recognized like B-DNA. (**a**) Gel-mobility shift titration for all types of complexes upon TFAM binding to G4-LSP22H (Fig. 2e). Fractions of unbound (triangles), singly bound (shift I, squares), and doubly bound (shift II, circles) DNA are represented. The solid curves result from fitting the data according to Eqs 3 (**b**) Model of binding of two TFAM molecules (in blue) to two sites on a tetramolecular G4. The intrinsic association constants, k_1_ and k_2_, represent the binding constants to G4 sites 1 and 2, while k_12_ is the cooperativity parameter representing the increased stability of protein-DNA complexes resulting from binding two protein molecules to the two G4 sites. (**c**) Intrinsic fluorescence emission spectra of TFAM (0.5 μM) in the absence (blue) and presence of 1.5 μM dsLSP22 (grey), G4-LSPas (violet) and G4-LSP22H (red) at an excitation wavelength of 275 nm.

We next characterized the double-shift pattern phenomenon. To this end, we used the tetramolecular G4-LSP22H, which rendered EMSAs of suitable quality. We used the fitting method established by Senear and Brenowitz^46^ for EMSA analysis of doubly-bound DNA. The titration points from the TFAM/G4-LSP22H EMSA were plotted as a fraction of total DNA for each shifted band and fitted to a curve (Fig. 3a), which was consistent with a doubly-bound DNA model (Fig. 3b). The best fit was obtained with microscopic equilibrium association constants k1 = 0.61 (±0.12)×10^–9^ M^−1^ and k2 = 0.69 (±0.25)×10^–9^ M^−1^, thus k1 ≈ k2, which indicates two independent and consecutive binding events on this G4-DNA^46^. The cooperativity parameter (*k*_*12*_) calculated from the fraction of singly-bound molecule was 0.58 (curve fitting) and 0.54 (Eq. 7), which indicates no cooperativity, or minor negative cooperativity. Therefore, TFAM binds with strong affinity to bi- or tetra-molecular G4s derived from mtDNA. The double shift pattern involves two proteins binding with almost equivalent probability to two potential binding sites on a tetra-molecular G4, the second TFAM binding event being independent from the first one. This sequential double-binding was not seen with B-DNA under identical experimental conditions.

TFAM contains two tryptophan residues per HMG-box, which allowed monitoring DNA binding by intrinsic tryptophan fluorescence quenching^47^,^48^. The crystal structures of TFAM in complex with B-DNA^32^,^48^,^49^ show that residues Trp88, Trp107 and Trp189 contact or are in close proximity to DNA, while Trp218 is buried in a small hydrophobic core within HMG2. Figure 3c shows that addition of dsLSP22 reduced the intrinsic fluorescence from the TFAM free-DNA sample by ~50%, which is consistent with previous reports^47^,^48^. In contrast, when TFAM was incubated with either G4-LSP22H or G4-LSPas, quenching increased up to ~70%, which indicates a different modification of tryptophan environment upon G4 binding as compared to B-DNA. This effect may be attributable to the above reported slightly higher affinity of TFAM for G4 substrates over dsDNA, and/or to a binding mode that differs between B- and G4-DNA.

**Figure 4:**
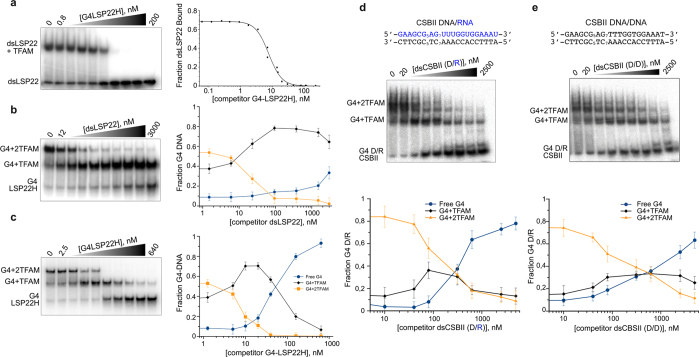
TFAM prefers G4 over dsDNA. **(a)** Competition assay by EMSAusing increasing amounts of unlabelled G4-LSP22H against ^32^P-labelled dsLSP22 (5 nM) in complex with TFAM (12 nM). The 0.8 and 200 labels correspond to initial and final concentrations (in nM) of G4-LSP22H. The fractions of bound DNA were plotted and fitted using Eq. 2. **(b)** Competition of unlabelled dsLSP22H against ^32^P-labelled G4-LSP22H (8 nM) in complex with TFAM (20 nM). The fraction of each species as a function of competitor addition is plotted on the right. (**c**) Homo-competition carried out as in (**b**), with ^32^P-labelled G4-LSP22H titrated with itself. **(d,e)** Competition assay using increasing amounts of unlabelled double-stranded CSBII, (**d**), a DNA/RNA heteroduplex or (**e**) a CSBII DNA homoduplex, against ^32-^P labelled CSBII G4-DNA/RNA (5 nM) in complex with TFAM (20 nM). The sequences of the competitors are indicated and the concentration ranged from 20 nM (lane 2) to 2.5 μM (lane 9).The fraction of each species as a function of competitor addition is showed at the bottom.

In addition to binding, we studied TFAM preference for G4 versus B-DNA by competition experiments. ^32^P-dsLSP22/TFAM complexes were firstly titrated with G4-LSP22H (Fig. 4a), which showed that a slight excess of G4 competitor (1.6-fold) was able to displace 50% of TFAM bound to B-DNA. Thus, the G4 competitor efficiently captured TFAM bound to dsDNA, in accordance with the difference in affinity and our pull-down experiments. We next performed the counter experiment and titrated ^32^P-labelled G4-LSP22H/TFAM complexes with dsLSP22 or unlabelled G4-LSP22H (Fig. 4b,c). In both cases the EMSAs showed a biphasic behaviour indicating sequential unbinding of the two TFAM molecules from the labelled G4s. However, the competition efficiency was markedly different. The homo-competition reaction (Fig. 4c) followed the expected pathway of bound-protein titration by the same substrate in the absence of cooperativity. 50% displacement of the doubly-bound complex and the converse 50% enrichment of the 1:1 complex were found for equimolar amounts of competitor and probe. This indicated the displacement of one TFAM from the doubly-bound complex and its binding to the unlabelled G4. Further titration with the competitor resulted in loss of singly-bound species concomitantly with the increase of the free probe, the latter reaching 50% for a 10x-fold excess of the competitor. Competitions with the dsDNA fragments showed the same pattern, but displacement of the singly-bound complexes was dramatically delayed. While the doubly-bound species were displaced as for the homo-competition, the singly-bound species were delayed, as expected for competition of a low-affinity substrate against two equivalent high affinity binding sites. A 3–4-fold excess of dsDNA was required to compete 50% of doubly-bound G4-DNA. The singly-bound species were not displaced even at a 375-fold excess of the competitor. Similar results were obtained when CSBII G4-RNA/DNA in complex with TFAM were titrated with CSBII dsDNA and a hetero-duplex RNA/DNA, the latter being a possible transcription intermediate. In the second case, however, competition was more ‘efficient’, with detection of around 80% of the free probe for a 500-fold excess of competitor, suggesting again a distinct behaviour of TFAM with RNA. These results confirm that TFAM binds with higher affinity to G4 than to the corresponding linear duplex DNA and therefore is displaced from B-DNA by G4-DNA.

**Figure 5:**
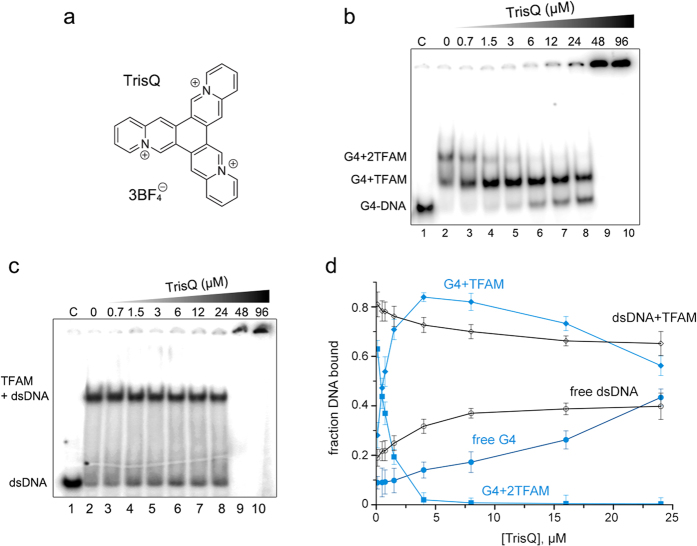
TrisQ selectively displaces TFAM from G4- over ds-DNA. (**a**) Structure of TrisQ. (**c,d**) EMSA showing the binding of TFAM (20 nM) to 8 nM of ^32^P-labelled G4LSP22H (**c**) or to ^32^P-labelled dsLSP22 (6 nM) (**d**), competed by the indicated amounts of TrisQ. “C” indicates the control sample of DNA without TFAM. (**d**) Quantification of the band shifts from panels (**b,c**), the points and fitting corresponding to the sample containing G4 are shown in blue.

Finally, we aimed at selectively displacing TFAM from G4 with a specific ligand. For this purpose we used a star-shape triazoniatrinaphthylene (TrisQ), which shows selectivity for tetramolecular G4- over B-DNA due to specific π-π interactions with the hydrophobic ring of terminal exposed G-quartets^50^. We undertook the same competition assays by adding increasing amounts of TrisQ to samples containing fixed amounts of TFAM/dsLSP22 or TFAM/G4-LSP22H complexes (Fig. 5). TrisQ addition to the mixture of singly and doubly bound TFAM/G4 complex resulted in biphasic competition similar to that of Fig. 4c, consisting of a rapid decrease of doubly-bound complexes followed by an accumulation of singly-bound ones. The latter were next progressively competed up to the release of free G4. 40% of free G4 was detected at 24 μM concentration of TrisQ. Above this concentration, the DNA aggregated (Fig. 5b, lanes 9–10). A similar aggregation at the same TrisQ concentration was observed for the dsDNA/TFAM complex (Fig. 5c, lanes 9–10). However, in this case TrisQ competed weakly with dsDNA-bound TFAM. This is consistent with the affinity of TrisQ for G4-DNA (in the μmolar range^50^), which was thus lower than that of TFAM and thus impaired effective displacement of the protein from the G4. Regarding the TFAM/dsDNA complex, TrisQ competition was inefficient, since free dsDNA increased only by about 10–15% before aggregation. Therefore, TrisQ specifically competed TFAM for binding to the G4-DNA rather than to dsDNA. In addition, this result evokes a competition between TFAM and TrisQ for the accessibility to the external G-quartets. This suggests hydrophobic stacking with residues of the HMG domains that are key determinants in TFAM binding to dsDNA^49^.

### G-quadruplex specificities required for TFAM binding

**Figure 6:**
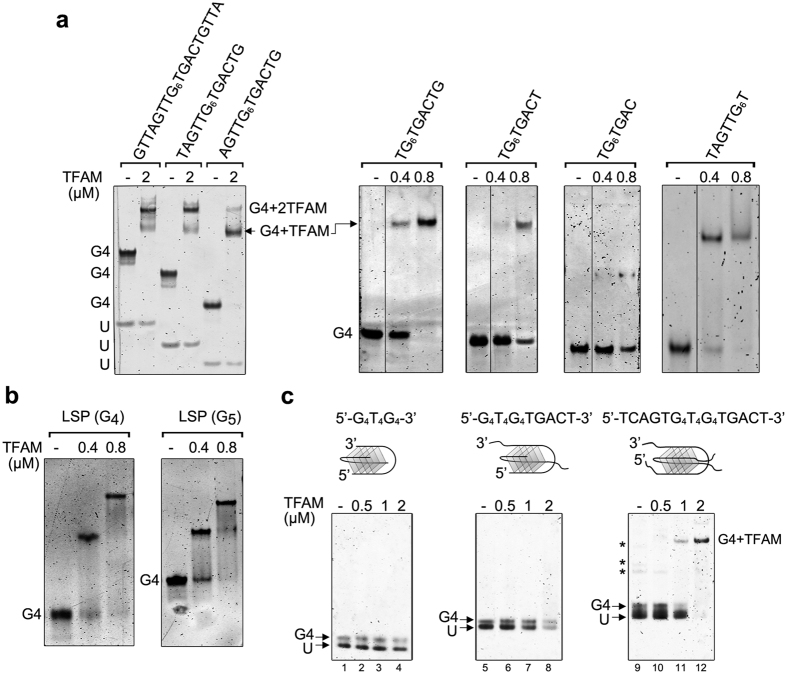
Binding specificities of TFAM to G4 DNA. **(a)** Examples of complexes obtained upon incubation of the indicated amounts of TFAM (in μM) with G4 tetramers of various lengths and sequences. (−) indicates absence of protein; (U), unfolded oligonucleotide. The gel on the left panel contains 0.5 μM G4-DNA/lane, the others contain 0.2 μM. (**b**) TFAM/G4 complexes obtained with tetramolecular G4 (0.2 μM) assembled from LSP sequence containing a tract of four (G4) and five (G5) guanines. (**c**) Complexes obtained between TFAM and bimolecular G4-DNAs formed by G_4_T_4_G_4_, G_4_T_4_G_4_TGACT and TCAG_4_T_4_G_4_TGACT oligonucleotides. The topologies of the bimolecular substrates are schematized above each gel, with folding involving diagonal loops here, although lateral loops can also exist. (−) indicates absence of protein. U: unfolded oligonucleotide. Each lane contains 0.8 μM of the DNA substrates (0.4 μM dimers). Lanes (2–4), (6–8) and (10–12) contain 0.5, 1 and 2 μM of TFAM, respectively. Asterisks for TCAG_4_T_4_G_4_TGACT indicate other G4 species such as parallel tetramers.

To further analyse the G4 determinants required for TFAM binding, we prepared G4s of different lengths and sequences and screened their recognition by EMSA. The experiments were performed with titrations at stoichiometric conditions using DNA fluorescent staining to easily discriminate between absence of binding, single and double-binding at protein:G4 ratios of 1:1, 2:1 and 4:1. We first shortened the 3′ and 5′ single-stranded (ss-) overhangs of tetramolecular G4-LSP22H in order to determine their involvement in the binding. We found the G4 (5′-TG_6_TGACT-3′)_4_ or (5′-TAGTTG_6_T-3′)_4_ as the minimal sequences to bind one TFAM (one single shift), with an apparent Kd of 5 nM for 5′-TG_6_TGACT-3′. Removal of one additional base cancelled protein binding (Fig. 6a). Reduction in the number of G-quartets from 6 to 4 did not modify TFAM binding (Fig. 6b). Presence of overhangs at both sides of the G-quartets barrel restored a double band-shift pattern (Fig. 6a). To better understand the role of the ss-overhangs, we next compared TFAM binding to tetramolecular G4 (TG_6_T)_4_ and to either dsDNA fragments of equivalent length (8 bp) or their derivatives extended with 8 nt ss-overhangs (Fig. S5). TFAM did not bind either the 8 bp DNA or the branched structures (Fig. S5a–c), which excludes recognition of the junction between the dsDNA and its ss-overhangs. A 10 bp duplex equipped with ss-overhangs of 10 nt was neither recognized (Fig. S5d), suggesting that TFAM needs a dsDNA annealed from end-to-end to anchor its HGM-box domains as in the crystal structure^32^,^47^,^48^,^49^. In contrast, a G4 (TG_6_T)_4_ core extended by 6 nt at the 3′ end (14 nt/strand, Fig. S5e) resulted in a stabilized TFAM/G4 complex, while double extension in both the 3′ and 5′ ends restored the double binding (Fig. S5f–g). Thus, TFAM appears to specifically recognize a G4 motif composed of a G-quartet core with 5 nt ss-overhangs. We next substituted two ss-overhangs by a short T_4_ loop, forming a bimolecular G4 with the G_4_T_4_G_4_ sequence (Fig. 6c). Similar to tetramolecular (TG_6_T)_4_, this G4 was not bound by TFAM, even if extended with a 3′-TGACT overhang. TFAM binding was recovered when the bimolecular G4 was extended with two TGACT overhangs (Fig. 6c), showing the need of at least two overhangs protruding from one side of the G-quartet barrel to stabilize protein binding. The presence of a T_4_ loop precluded the double-binding. The key role of ss-overhangs was confirmed by extending the length of intramolecular c-myc and ILPR G4 with ss-overhangs of at least 5 nt (see Table 1 and Fig. S4c,d). This produced two ss-overhangs on the same side of parallel ILPR G4, while antiparallel c-myc presented only one ss-overhang. The apparent Kd for the extended ILPR G4 was around 26 nM, 4.5-fold higher than for former ILPR. TFAM binding to the extended c-myc was similar to un-modified c-myc. In conclusion, these and above experiments show a specific binding of TFAM to G4s bearing a core of stacked G-quartets elongated with at least two ss-overhangs of 5 nt each on the same side. A G4 presenting ss-overhangs of sufficient length on both sides of the stacked G-quartets can load two TFAM molecules. These results indicate that TFAM binding to G4 involves the ss-overhangs, the terminal G-quartets and/or their junction. DNA duplex of the same nt length and with or without ss-overhangs are not bound under the same conditions.

### Molecular determinants of TFAM for G4 binding

**Figure 7:**
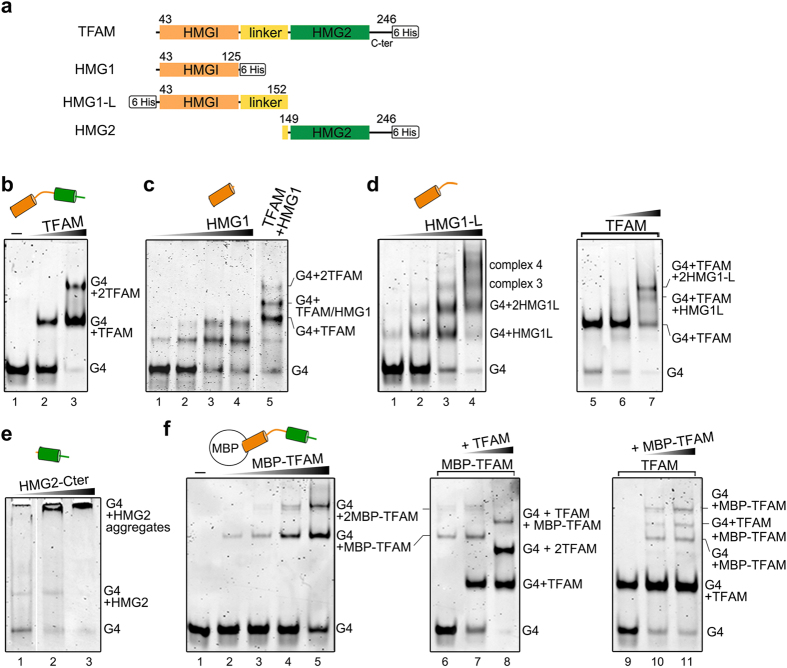
Domains of TFAM involved in G4 binding. **(a)** Diagram of TFAM domains and TFAM constructs used in this study. **(b–f)** Binding to G4-LSP22H (0.2 μM) followed by EMSA. (**b**) Binding of 0.4–0.8 μM TFAM (lanes 2–3); **(c)** binding of 0.32, 0.75, 1.5, and 3 μM HMG1 (lanes 1–4) or a mixture of 0.6 μM TFAM and HMG1 (lane 5). **(d)** Left panel: complexes obtained with 0.32, 0.75, 1.5, and 3 μM HMG1-L (lanes 1–4). Right panel: TFAM (0.5 μM)/G4-LSP22H complexes (lane 5) were preformed 10 minutes before the addition of 0.5 or 1 μM of HMG1-L (lanes 6–7). **(e)** Binding of 0.75, 1.5 and 3 μM HMG2-Cter. **(f)** Left panel, binding of 0.25 to 2 μM MBP-TFAM (lanes 2–5). Central panel, MBP-TFAM (0.8 μM)/G4-LSP22H complexes (lane 6) were preformed 10 minutes before the addition of 0.5–1 μM of TFAM (lanes 7–8). Right panel: preformed TFAM (0.6 μM)/G4-LSP22H complexes mixed with to 0.8 and 1.6 μM MBP-TFAM (lanes 10–11). For clarity, one free G4 migration control is shown in lanes 1 of panels (**b**,**f**).

To assess the TFAM domains involved in G4-LSP22H recognition, we produced the following TFAM constructs (Fig. 7a): HMG1 domain alone (HMG1, from Ser43 to Gln125); HMG1 domain extended with 27 residues from the linker (HMG1-L, Ser43-Leu152); and the HMG2 domain with the C-terminal tail (HMG2-Cter, Leu149-Cys246). These constructs were tested for binding, individually or in combination with full-length TFAM, by EMSA. HMG1, the dominant dsDNA-binding domain^42^,^47^, shifted the G4 twice, like full-length TFAM (Fig. 7c). A 1:1 mixture of TFAM and HMG1, incubated with G4-LSP22H, yielded an additional band with intermediate mobility, below the double-shift observed for the full-length protein. This indicates heterogeneous binding of one TFAM and one HMG1 molecule to one G4-DNA (Fig. 7c, lane 5). Surprisingly, HMG1-L formed up to four distinct up-shifted complexes with the G4 (Fig. 7d), suggesting stabilizing contacts by the basic linker. This linker has been shown to increase the dsDNA binding ability of HMG1^47^ and to interact with the dsDNA narrow groove in the crystallographic U-turns^32^,^48^,^49^. According to the available three-dimensional structures, the G4 substrate likely present four identical grooves of width compatible with linker dimensions. Thus, four up-shifts suggest the binding of four HMG1-L per G4 through insertion of four linkers into these grooves. This would orientate the protein in the longitudinal axis of the G4 and position the HMG domains on the terminal G-quartets and overhangs. Accordingly, the addition of HMG1-L to preformed 1:1 G4/TFAM complexes (Fig. 7d, lanes 5–7) led to the formation of two additional up-shifts (lane 7), presumably one TFAM and one (or two for the highest band in lane 7) HMG1-L molecule on one G4. HMG2 does not bind dsDNA^29^,^42^ efficiently. It associated here with the G4 substrate but in the form of a band of weak intensity migrating like the G4/HMG1 complex, forming both smearing and high molecular mass species that did not enter the gel (Fig. 7e). Finally, TFAM double binding on G4s was further confirmed by generating a fusion between TFAM and the maltose-binding protein (MBP-TFAM), which also shifted the G4-DNA twice (Fig. 7e). When both TFAM and MBP-TFAM were mixed, an intermediate band-shift appeared, corresponding to a heterogeneous complex of one MBP-TFAM and one TFAM molecule bound to one G4 (lanes 8, 10, 11), similar to the aforementioned combinations of full-length and protein domains.

## Discussion

Recent studies have proposed that the particular enrichment of G-rich sequences in mtDNA causes the formation of G-quadruplexes. An RNA/DNA G4 at CSBII^20^,^21^,^22^ suggests a functional role in contributing to R-loop stabilization, similar to that observed in nuclear DNA^51^. Numerous additional G4s are predicted along the mtDNA sequence^15^,^16^,^17^ and may form during replication, leading to genomic instability^52^,^53^. These potentially G4-forming positions correlate with deletion hotspots in mtDNA^16^,^17^,^23^,^54^, which are associated with disparate forms of disease. The tendency of mtDNA to form G4 in cells^15^,^16^,^17^,^18^ raises the obvious question of mitochondrial factors capable of recognizing these structures. We provide evidence that human TFAM, the major nucleoid component and essential factor for mtDNA transcription and maintenance, strongly binds to diverse G4 structures *in vitro*. We show that TFAM presents an affinity to tetra- or bi-molecular G4 similar than to the corresponding B-DNA, which in all cases is an inefficient competitor. In addition, our pull-down experiments demonstrate that, even in the presence of mtDNA and DNA competitors, the G4 baits systematically captured TFAM. Taking into account that TFAM coats the entire mtDNA, these findings suggest that any G4 structure that could form in mtDNA, even transiently, should be bound by TFAM.

HMG-box proteins are remarkably versatile DNA-binding molecules, involved in a wide array of functions, including transcription, replication and chromatin architecture regulation^28^,^30^. To our knowledge, this is the first report of G4 recognition by a protein of the HMG-box family *in vitro*. This binding is of nanomolar affinity for model mitochondrial sequences assembled into tetramolecular (LSP) or bimolecular (CSBII) G4s. The mechanism of transcription termination at CSBII is protein-independent *in vitro*^22^,^55^. Thus, probably TFAM is not involved in such termination events. However, long RNAs are found to be bound to mtDNA where they may restrict mtDNA supercoiling^56^ or be related to replication^57^. These RNA/DNA hybrids could induce bimolecular G4s similar to CSBII and be a substrate for TFAM. On the other hand, TFAM binding to nuclear intra-molecular G4s was weaker and dependent on G4 length and topology. In the nucleus, intra-molecular G4s are mostly found in alternative promoters and in telomeres, and these are absent in mtDNA.

Our results indicate that TFAM/G4 interactions involve elements beyond the G4 core. This is common in G4-binding proteins, such as nucleolin that recognizes the G4 backbone and/or loops^10^; the Fragile X Mental Retardation Protein that recognizes the duplex region, the duplex–quadruplex junction, and a mixed tetrad of an mRNA G4 element^11^; ss-overhangs are part of the recognition motif in HIV-1 NCp7^12^ or in gene 5 protein of Fd phage^58^. Additionally, our data suggest a binding model. First, TFAM does not bind to unstructured oligonucleotides (here and ref. 33), nor two short dsDNAs with or without ss-overhangs, but specifically to G4s that present at least two overhangs on the same side of the G4 core. Further, our competition experiments with TrisQ point to a key interaction at the hydrophobic surface of an external G-quartet plateau. Thus, the combination of both the G-quartets and ss-overhangs are required for recognition, which could also involve the G-quartet/ss-overhangs junction. A barrel of G-quartets bearing ss-overhangs at both sides could bind two TFAM molecules independently. Further, the binding of TFAM domains suggests positioning along the main axis of the G4-DNA, e.g. by insertion of the linker into the grooves. This arrangement would localize the HMG domains close to both terminal G-quartets and their ss-overhangs. There, insertion of hydrophobic residues between stacked base pairs could occur as in B-DNA^29^. Visual inspection of TFAM and G4 structures confirms that two TFAM molecules fit on opposite faces along the G4 longitudinal axis (Fig. 3b). This simple binding mechanism would enable a great adaptability to recognize the majority of G4 topologies, independently of strand orientation or composition.

A surprisingly complex pattern of ladder-like shifted bands was observed with CSBII RNA, RNA/DNA G4 and duplex/G4 structures. This result suggests a new G4-dependent TFAM-TFAM interaction that leads to protein multimerization beyond dimerization^32^,^42^. Interestingly, a similar G4-driven oligomerization has been observed for yeast chromatin-binding protein Rif1^59^. This behaviour suggests a mechanism distinct from simple G4 capping. Formation of high MW species in presence of the HMG2 domain, as well as the increase of tryptophan quenching with respect to dsDNA suggest additional interactions of this domain with the G4 and/or a conformational change upon G4 binding. A G4-dependent conformational transition of TFAM, different from the one occurring with B-DNA, is an attractive hypothesis to pinpoint a signalling role upon G4 binding. For example, a G4-induced TFAM conformation could signal presence of G4s to other mitochondrial factors including G4-specific helicases. In this context, the reported interaction of TFAM with the mitochondrial RECQ4L helicase^39^ evokes a potential partnership. TFAM binding to the CSBII G-rich sequence, and especially the high affinity and TFAM oligomerization observed with the RNA/DNA G4 engaged in R-loop stabilization^20^,^21^ is also attractive regarding a potential functional role at this position. In addition, a G_6_ stretch is found in both a H-strand sequence recognized by LON and TFAM *in vivo*, and both proteins bind to the G4 formed by this sequence *in vitro*. TFAM bound to dsLSP is protected from proteolysis by LON, which also occurs in the presence of LSPas^37^. Our results suggest that this latter unexplained protection arises from the binding of TFAM to the G4 form of LSPas. The trio of TFAM, LON and G4-DNA offers exciting prospects for a functional mechanism underlying TFAM binding to either G4-DNA or dsDNA, which could be regulated by LON and related to a cellular checkpoint mechanism that would modulate mtDNA maintenance and/or other events.

## Methods

### Recombinant TFAM and G4 production

A C-terminal 6-histidine tagged mature human TFAM (residues 43–246; UniProt Q00059), and shorter constructs HMG1 and HMG2-Cter were produced as described^49^. N-terminal His tagged HMG1-L was cloned using In-Fusion^TM^ in a pOPINF vector, and likewise purified. MBP-TFAM fusion protein was cloned using vector pETM40 and expressed in Rossetta2 cells, which were lysed in 750 mM NaCl, 50 mM HEPES pH 7.5 and 1 mM DTT. The fusion protein was purified using a dextran-sepharose column (MBPTrap HP 1 mL GE Healthcare) in 750 mM NaCl, 50 mM HEPES pH 7.5, 5 mM DTT and 10 mM maltose as elution buffer. TFAM and the constructs were concentrated to 5 mg/mL in 50 mM HEPES pH 7.5, 750 mM NaCl, 1 mM DTT and 10% glycerol, and stored at −80 °C.

### Nucleic Acids

Oligonucleotides were purchased from Sigma-Aldrich. Unless otherwise indicated, DNAs and RNA G4s were generated by oligonucleotide suspension at a concentration of 5 mM in 10 mM sodium cacodylate pH 6.5 and 100 mM KCl. DNA was quantified by measuring the absorbance at 260 nm (A_260_) immediately after oligonucleotide suspension. Tetramolecular G4s were incubated for 5 min at 95 °C, then cooled and stored at 25 °C for at least one week. Intramolecular G4 for c-myc and ILPR were assembled at a concentration of 400 μM, and immediately chilled on ice after incubation at 95 °C. The CSBII G4 RNA-DNA hybrid form was produced by incubating 10 μM of RNA and 40 μM of DNA CSBII HS oligonucleotides in a buffer previously described in^20^, containing 50 mM TrisHCl pH 8.0, 10 mM NaCl, 6 mM MgCl_2_, 1 mM CaCl_2_ and 1 mM EDTA. The oligonucleotides were heated for 5 min at 95 °C and incubated overnight at 37 °C. G4 folding was assessed over time by native PAGE at room temperature in 0.5× TBE, followed by staining with SybrGold (Life Technologies). DsDNA fragments were formed by heat annealing of equimolar amounts of oligonucleotides in 20 mM TrisHCl pH 7.5, 100 mM NaCl and 5 mM MgCl_2_.The CSBII and LSP22 dsDNA were purified from contaminating G4s by gel extraction and ethanol precipitation. G4 denaturation was performed by 30 min incubation at 37 °C in 25 mM NaOH followed by neutralisation at pH 7. Molar amounts of G4-DNA refer to moles of four-stranded structures (1 mol of tetramer = 4 mol of single strands; 1 mol of dimer = 2 mol of single strands). 4a,10a,16a-triazoniatrinaphthylene (TrisQ) tetrafluoroborate was synthesized as described elsewhere^50^, and solubilized at a concentration of 2 mM in milliQ water.

### Cell extract preparation

Mitochondrial fractions were obtained by differential centrifugation of HeLa cell homogenates. HeLa cells were cultured in DMEM (Gibco) supplemented with 10% (v/v) foetal bovine serum, 10 mM HEPES pH7.4 and 1% (v/v) antibiotics (10,000 U/mL penicillin, 10 mg/mL streptomycin). Cells were grown at 37 °C in a humidified atmosphere with 5% CO_2_, washed in ice-cold PBS 1x, scraped and lysed with a syringe in mitochondrial extraction buffer (250 mM sucrose, 10 mM TrisHCl pH 7.4, 1 mM EDTA, 1 mM sodium orthovanadate, 50 mM NaF, 5 mM sodium pyrophosphate and protease inhibitor cocktail) and centrifuged at 1000 × g for 10 min at 4 °C. The supernatant containing mitochondria was then centrifuged at 13,000 × g for 10 min at 4 °C. The mitochondria-enriched pellet was dissolved in mitochondrial extraction buffer containing a cocktail of protease and phosphatase inhibitors (Roche) and centrifuged at 13,000 × g for 10 min at 4 °C. The pellet was then suspended in 30–50 μL of mitochondrial extraction buffer without inhibitors (5 times the volume of the precipitate). All fractions were stored at −80 °C.

### Pull-down assay

2 mg of Streptavidin-coated magnetic beads (Dynabeads M280, Invitrogen) were functionalized by overnight incubation at 4 °C of 30 μg of biotinylated

CSBII G4-DNA (biotin-5′-GAAGCG_5_AG_7_TTTGGTGGAAAT) or a CSBII dsDNA fragment produced from annealing with the complementary oligonucleotide (5′-ATTTCCACCAAAC_7_TC_5_GCTTC) in 50 mM TrisHCl (pH 7.7), 0.5 mM EDTA and 1 M NaCl. To lower non-specific TFAM binding, the beads were vigorously shaken for 15 min at 25 °C in the presence of a 2-fold dilution of 1X blocking buffer containing 20 mM TrisHCl pH 7.5, 10 mM HEPES pH 7.2, 4.4 mM EDTA, 9% sucrose, 110 mM NaCl, 5 mM MgCl_2_, 12% glycerol, 8 mM DTT, 0.02% (v/v) Tween20 (PanReacAppliChem), 0.1 mg/mL salmon sperm DNA (Sigma-Aldrich) and 0.5 mg/mL bovine serum albumin (Sigma-Aldrich). 100μl of mitochondrial fractions were lysed with 100μl of a 2X binding buffer containing 40 mM TrisHCl pH 7.5, 0.2 mM EDTA, 100 mM NaCl, 10% glycerol, 1% Triton X-100, 2X protease inhibitor cocktail (Roche). 20μl of this mixture were mixed with the beads, supplemented with 25 μL of the 1X blocking buffer. The binding mixture was then incubated for 40 min at 25 °C. Beads were pelleted using a Magna-Sep^TM^ magnetic particle separator (Invitrogen) and washed with 25 μL of buffer (20 mM TrisHCl pH 7.5, 0.1 mM EDTA, 50 mM NaCl, 5% glycerol, 0.1% Triton X-100, 4 mM DTT). DNA-bound proteins were eluted by stepwise incubations in 150 μL of buffer (25 mM TrisHCl pH 7.5, 1x EDTA-free protease inhibitor, 4 mM DTT), containing increasing concentrations of NaCl. The collected fractions were precipitated with cold acetone at −80 °C and loaded on Tris-Glycine 15% SDS-PAGE before Western blotting. The pull-down assay with recombinant TFAM was undertaken in the same conditions, using 300 ng of recombinant TFAM. Anti-TFAM antibodies (1/1000,Abcam) and anti-porin (1/5000,Calbiochem) were used for western blots. Peroxidase-conjugated anti-mouse and anti-rabbit immunoglobulins (Santa Cruz biotechnology) were used as secondary antibodies. Membranes were developed by an ECL Western blot analysis system (Amersham).

### Mobility shift assays

The binding assays were carried out with 5′-^32^P labelled DNA probes or unlabelled DNA fragments stained with SybrGold. G4s were labelled using [γ-^32^P]ATP (Perkin Elmer) and T4 polynucleotide kinase and purified in G25 columns (GE Healthcare). A total of 500 fmol of DNA was incubated with the indicated concentrations of TFAM in 10 μL reactions, containing 25 mM TrisHCl pH 7.0, 90 mM NaCl, 1 mM DTT, 3% glycerol and 0.01% Tween20 for 30 min at room temperature. Serial protein dilutions were performed on ice in 5% glycerol, 700 mM NaCl, 20 mM TrisHCl pH 7.0, 1 mM DTT and 0.01% Tween20. Protein titrations for Kd evaluation were carried out by serial dilutions of TFAM in fifteen points, repeated three times for each DNA substrate. The same protocol was used for non-radioactive experiments with 0.2–0.5 μM of unlabelled DNA per reaction. Binding reactions were loaded onto 10% polyacrylamide gels in 0.35x Tris-Borate EDTA buffer and electrophoresis was run at 11 V/cm at room temperature in a MiniVE apparatus (Hoeffer). Radiolabelled gels were dried, exposed to a phosphoimager screen and quantified with a Typhoon 8600 (Molecular Dynamics). Gels stained with SybrGold were digitalized with the same apparatus in the appropriate mode.

#### Model of Protein-DNA interaction

The titration of DNA bands was described by a modified form of the Hill equation (1), that can compensate for deviations from ideal conditions, including incomplete binding caused by loss of protein sample at low concentrations, cooperative binding, oligomerization, or other more complex mechanisms of binding,


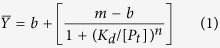


where 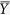 is the fractional saturation at the site, *P*_*t*_ is the total protein concentration used in the equilibration and *K*_*d*_ the equilibrium dissociation constant, *m* and *b* are normalization factors that represent the fraction of bound DNA at the upper and lower asymptotes of the titration and *n* is the Hill coefficient. The Hill coefficient measures the cooperativity of binding, and for bimolecular association of protein and DNA its value is 1. Deviations from unity may indicate cooperative binding of multiple proteins or binding reactions that have not reached equilibrium. Small deviations from integer values are commonly caused by the adherence of protein or DNA to the equilibration vessel. This phenomenon occurred with TFAM, which, in addition, aggregated around the 500 nM-1 μM range, both effects being suppressed to some extent by the addition of 0.01% v/v. Tween 20 to the reaction buffer. The competition reactions of Fig. 4a were plotted as the fraction of bound labelled DNA versus the concentration of unlabelled competitor and the data were fitted to a sigmoidal dose-response curve (2).


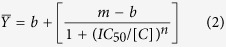


where *C* is the concentration of unlabelled competitor^60^.

To examine the interactions of TFAM with the G4, we assumed that the protein sequentially filled two binding sites on the G4-DNA (Fig. 3b) and used the curve-fitting method of Senear and Brenowitz for a two-sites system analysis by EMSA^46^. This method provides the relative fraction of DNA molecules with an *i* number of ligands bound, thus *i* = 0, 1 or 2, and 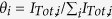 where *I* is the intensity of the number of counts per band measured with the phosphorimager and the summation is over all of the bands in a given lane *i*. The binding equation for the 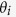
yields:


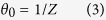



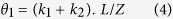



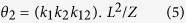


where *Z* is the binding polynomial equal to 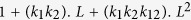. *k*_1_ and *k*_2_ are microscopic equilibrium association constants for intrinsic binding to sites 1 and 2; *k*_12_ is the constant describing cooperative interactions during binding of the second site. An alternative estimate of the cooperativity parameter was made by measuring *θ*_1*max*_ the maximum value of *θ*_1_.


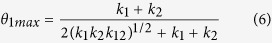


If *k*_1_is equal to *k*_2_, *k*_12_ can be written


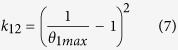


Therefore, if *θ*_1*max*_ = 0.5, then *k*_12_ = 1, indicating no binding cooperativity. Lower values of *θ*_1*max*_ indicate positive cooperativity, and higher values indicate negative cooperativity.

#### Intrinsic fluorescence quenching

Fluorescence intensities of TFAM tryptophans were measured on an Aminco-Bowman AB2 spectrofluormeter at 25 °C in buffer containing 0.25 μM TFAM in 25 mM TrisHCl pH 7.5, 150 mM KCl and 1 mM DTT. Scans were recorded using an excitation wavelength of 275 nm and an emission wavelength ranging from 300 to 400 nm. Slit widths for both excitation and emission were kept at 4 nm, with a photomultiplier voltage of 750 V.

## Additional Information

**How to cite this article**: Lyonnais, S. *et al*. The human mitochondrial transcription factor A is a versatile G-quadruplex binding protein. *Sci. Rep.* **7**, 43992; doi: 10.1038/srep43992 (2017).

**Publisher's note:** Springer Nature remains neutral with regard to jurisdictional claims in published maps and institutional affiliations.

## Supplementary Material

Supplementary Information

## Figures and Tables

**Table 1:**
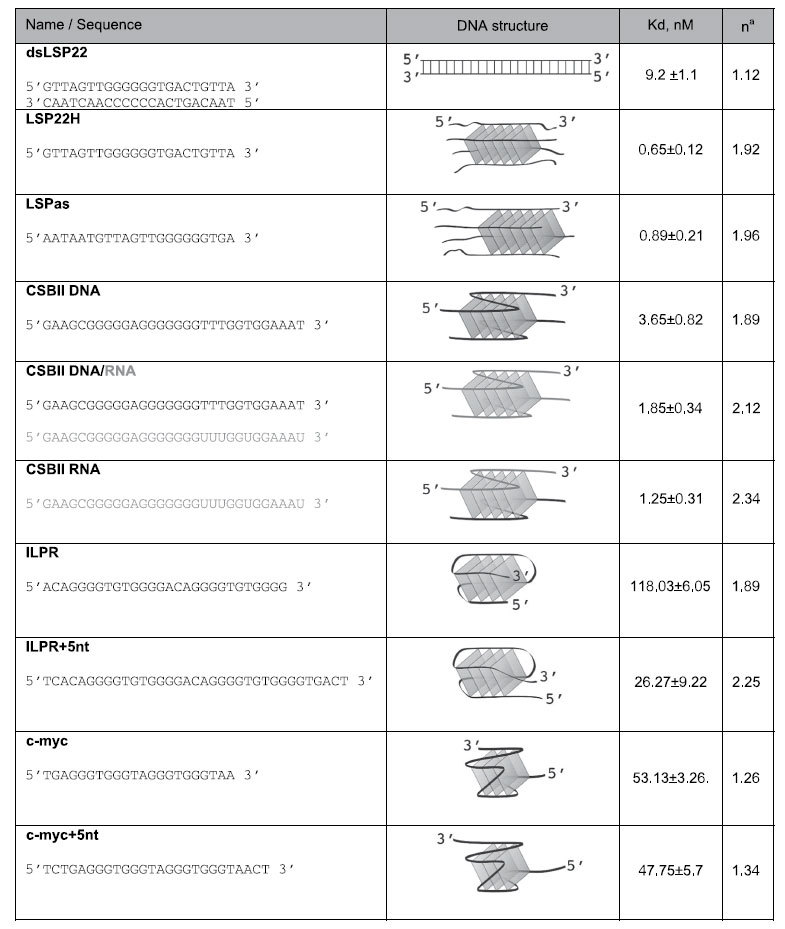
Nucleic Acids bound by TFAM and quantitative comparison.

Only sequences whose G4 structures are not represented in any of the figure panels are included. See ref. 22 for the 22bp-long three-stranded DNA-RNA construct structure. n^a^ is an estimate of the stoichiometry derived from fitting of the EMSA titration to Eq. (1). RNA strands are coloured in grey. The tetramolecular and bimolecular G4 contain strands in parallel orientation, but only the orientation of one strand is indicated for clarity.
