# Corrigendum: The human mitochondrial transcription factor A is a versatile G-quadruplex binding protein

**DOI:** 10.1038/srep45948

**Published:** 2017-04-06

**Authors:** Sébastien Lyonnais, Aleix Tarrés-Solé, Anna Rubio-Cosials, Anna Cuppari, Reicy Brito, Joaquim Jaumot, Raimundo Gargallo, Marta Vilaseca, Cristina Silva, Anton Granzhan, Marie-Paule Teulade-Fichou, Ramon Eritja, Maria Solà

Scientific Reports 7: Article number: 4399210.1038/srep43992; published online: 03 09 2017; updated: 04 06 2017

The original version of this Article contained an error in the spelling of the author Aleix Tarrés-Solé, which was incorrectly given as Aleix Tarrés-Soler. This error has now been corrected in the PDF and HTML versions of the Article.

