# Supplementary material for: The human mitochondrial transcription factor A is a versatile G-quadruplex binding protein
Source: Sci Rep. 2017 Mar 9;7:43992. doi: 10.1038/srep43992 (PMC5343656; doi:10.1038/srep43992)
Supplement: Supplementary Information [file srep43992-s1.pdf]

Supplementary Information

The human mitochondrial transcription factor A is a versatile G-quadruplex binding protein

Sébastien Lyonnais[1,a)], Aleix Tarrés[1], Anna Rubio-Cosials[1],Anna Cuppari[1], Reicy Brito[1], Joaquim Jaumot[2], Raimundo Gargallo[2], Marta Vilaseca[3], Cristina Silva[1], Anton Granzhan[4], Marie-Paule Teulade-Fichou[4], Ramon Eritja[5], and Maria Solà[1,b)]

[1] *Structural MitoLab, Department of Structural Biology, Molecular Biology Institute of Barcelona (CSIC), Barcelona, 08028, Spain*

[2] *Department of Analytical Chemistry, University of Barcelona, Barcelona, 08028, Spain*

[3] *Mass Spectrometry Core Facility, Institute for Research in Biomedicine, IRB Barcelona, 08028 Barcelona, Spain*

[4] *UMR176 CNRS, Institut Curie, Centre Universitaire Paris XI, Orsay, France.*

[5] *IQAC-CSIC, CIBER-BBN, Barcelona, E-08034, Spain*

a) slycri@ibmb.csic.es

b) maria.sola@ibmb.csic.es

Supplementary Methods

Supplementary Figure S1

Supplementary Figure S2

Supplementary Figure S3

Supplementary Figure S4

Supplementary Figure S5

Supplementary references

Supplementary Methods

Circular Dichroism. CD spectra were recorded with a Jasco J-810 spectropolarimeter using 1 cm path length quartz cuvettes in a volume of 500µL containing 5µM DNA in 10mM sodium cacodylate pH 6.8 and 50mM KCl. Scans were performed at 25°C over a wavelength range of 220-320 nm with a scanning speed of 50 nm.min^{-1}, a response time of 4s, 1 nm pitch and 1 nm bandwidth. For each sample, the CD spectrum of buffer was recorded, subsequently the sample was added into the same cuvette, and the blank substracted from the collected data.

Mass Spectrometry. ESI-IM-MS experiments were performed on a Synapt HDMS G1 instrument (Waters, Manchester, UK) equipped with electrospray ionization, a travelling wave ion mobility cell and a time-of-flight mass analyzer. The instrument was used in negative electrospray ionization and ion mobility mode. The capillary voltage was set to -2.4V; cone voltage to 45V, extraction cone to 5V; source pressure (pirani reading) to 5.62mbar; source and desolvation temperatures were 100ºC and 200ºC, respectively; trap and transfer voltages were set to 10V. The ion mobility cell was filled with N_2 at 0.467mbar (pirani reading), and an electric field was applied to the cell in form of waves (wave height, 8V) that pass through the cell at 300 m/s. The bias voltage for ion introduction into the IM cell was 15V. External MS calibration was performed over 500-8000m/z using CsI. G4-LPS22H (1mM strand concentration) were prepared in 10mM sodium cacodylate pH 6.8, 150mM KCl, incubated one week at room temperature and dialyzed five times against 150 mM ammonium acetate pH 6.9 at 4°C. TG_6TGACT (1,2mM strand concentration) were prepared in 150mM ammonium acetate pH 6.9 and incubated two weeks at room temperature. DNA samples we diluted in 150mM ammonium acetate pH 6.9 to a 150 µM concentration of G4-DNA for ESI-IM-MS analysis.

Supplementary Figures

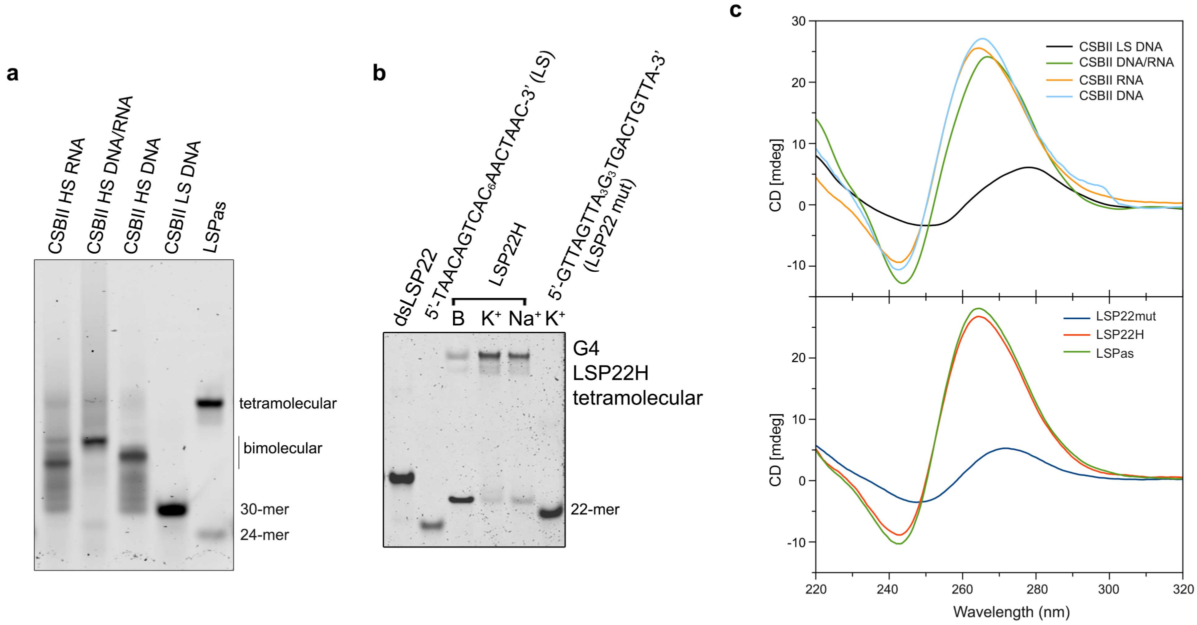

Supplementary Figure S1. mtDNA-G4 assembly and characterization. (a) PAGE separation of CSBII and LSPas oligonucleotides following G4 growth (see Table 1). The CSBII light strand (LS) DNA (Figure 4e) was used as migration control. Molecularity of the species is indicated on the gel. A prolonged (one week) incubation of CSBII (HS) DNA and RNA in 100 mM potassium yielded to complex mixtures of species, including however a major up-shifted band suggesting bimolecular G4. Incubation of RNA and DNA HS yielded more homogeneously to a band also migrating as a bimolecular G4, as seen previously (1). LSPas showed a homogeneous band, up-shifted band characteristic of a tetramolecular G4 (2). CD and EMSA experiments were performed with the G4 structures identified as bimolecular, after purification from preparative gel. **(b)** PAGE showing the formation of tetramolecular G4 for LSP22H in presence of 100 mM KCl or 100mM NaCl. (B) denatured G4 after prolonged boiling. Lane 1, duplex dsLSP22 used for figure 2; lane 2, LS sequence incubated in the conditions used for G4 formation. A mutated LSP sequence (LSP22mut), with substitution of three adenines in the G_6 stretch, was used as a migration control of the unfolded oligonucleotide. (**c**) CD Spectra in 50mM KCl of the indicated G4 and oligonucleotide controls (CSB II LS and LSP22mut). The CD spectra ascertain the formation of G4 structures, with strong positive ellipticity maximum around 265 nm and negative ellipticity minimum at 245 nm, the signature of parallel G4.

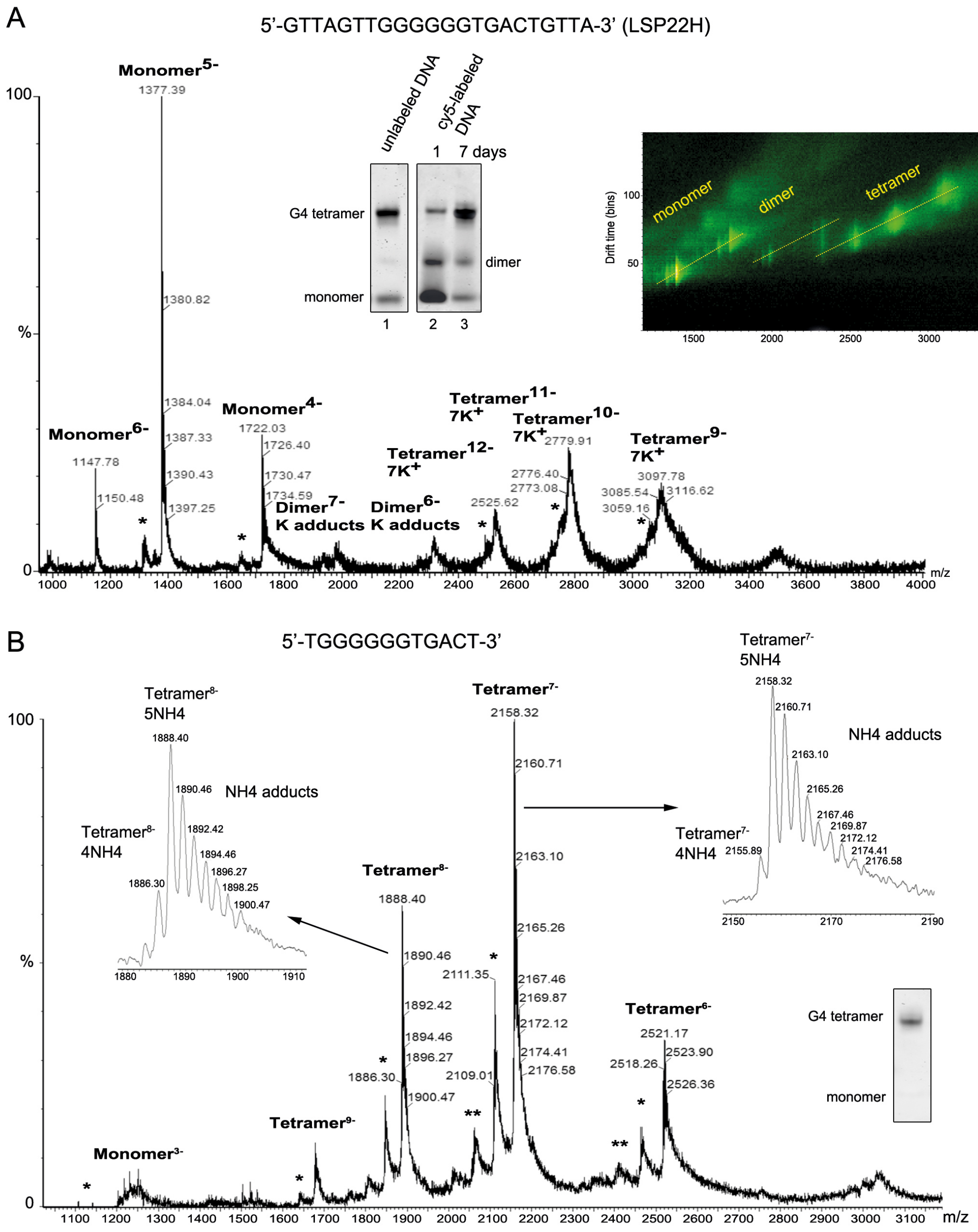

Supplementary Figure S2. Identification of tetramolecular G4 LSP species by electrospray mass spectrometry in negative mode. (a) ESI-MS spectrum of LSP22H after G4-DNA formation in KCl and extensive dialysis against 0.15M ammonium acetate. Inserts show SybrGold stained 12% PAGE of the LSP22H species before MS (lane 1). Insert on the right side shows the 2D ESI-MS and drift time distribution of the DNA species. The experiment shows the presence of monomer in three charge states (6-, 5- and 4-) and tetramer in four charge states (12-, 11-, 10- and 9-), although the distribution between monomers and tetramers was inverted compared with the PAGE separation. The tetramers remained associated with potassium ions, which indicates the absence of exchange between K^+ and

$NH4^+$, a reported phenomenon in the field of G-quadruplex (3). The wide peaks for the tetramer gives poor accuracy in the exact mass determination, and seven K^+ ions instead of the expected number of 5 are seen associated with the G4 structure. This perhaps attests for the presence of additional G-quartets, since LSP22H contains six other guanines residues, or the presence of K^+ on external G-quartets. Another unanticipated result is the detection of few amounts of dimeric species (D=13845± 12.93) associated with K^+, curiously poorly detected with SybrGold staining after gel electrophoresis (insert). This bimolecular population was however detected when we separated by PAGE a Cy5-5' end labeled LSP22H (insert) perhaps because of a stabilization induced by the Cy5 moiety. These dimers disappeared to the benefit of the tetramer upon prolonged incubation time and might be intermediates leading to the formation of the tetrameric structure, or "dead ends" products, as noted with other sequences containing long guanine stretches (4,5). **(b)** ESI-MS spectrum of TG_6TGACT after G4-DNA formation in 0.15M ammonium acetate. Peaks from 1880 to 1910 and 2150 to 2190Da were magnified on the full spectrum. The zooms clarify the nature of the cation adducts on the tetramer. Asterisks indicate impurities in the monomer or multimers incorporating one of these modified monomers. The insert on the right side shows the DNA species separated by PAGE before injection. The expected tetramolecular G-quadruplex contains up to five ammonium ions, which indicates that the structure contains six stacked G-quartets. No dimers were detected in this case, indicating that the dimers observed with LSP22H are probably stabilized by base pairing between the ss-overhangs or only formed in presence of K^+.

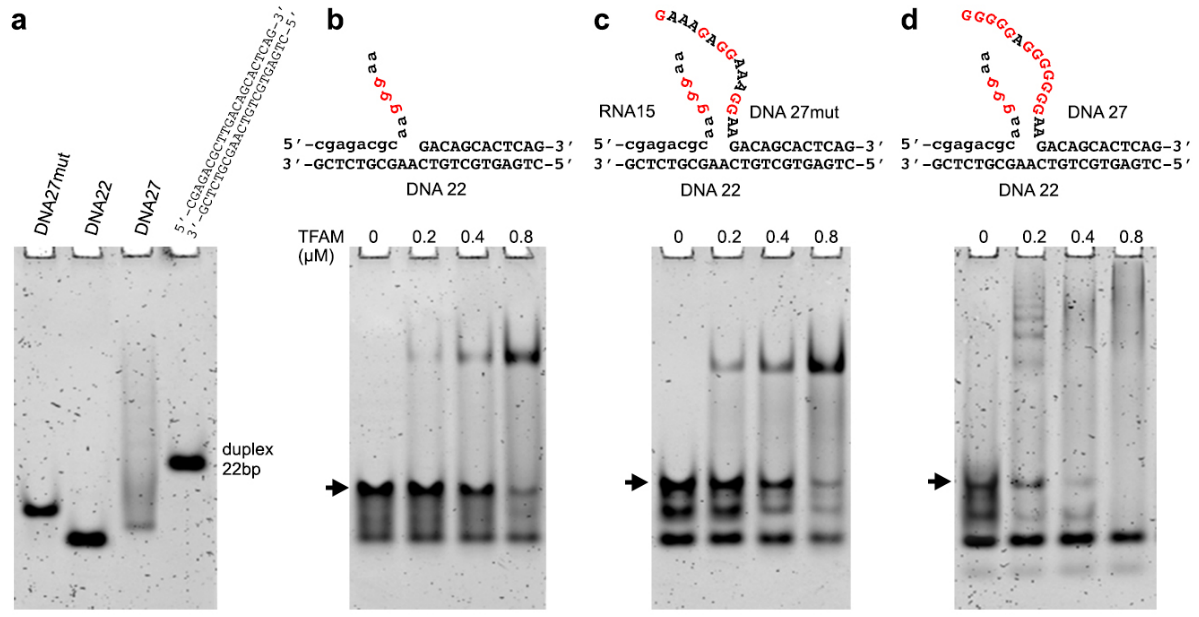

Supplementary Figure S3. TFAM association to duplex/G4 DNA constructs. Three stranded DNA/RNA substrates spanning the CSBII sequence were adapted from the constructs described in (6) and tested for TFAM binding. The gel and the binding buffer were complemented with 1mM $MgCl_2$ to stabilize the constructs, which were seen rather unstable due to the low amount of base-pairs. The gel on panel (**a**) indicates the mobility of the DNA oligonucleotides and the mobility of a full 22bp DNA duplex. Panels (**b**) to (**d**) show a 15mer H-strand RNA represented in lowercase, the two other strands are DNA. Reactive guanines are highlighted in red. In panel (**d**) the overhangs from RNA15 and DNA27 oligonucleotides were previously seen to form an hybrid RNA/DNA G4 (reference 6). The correctly annealed three-stranded structures are indicated by an arrow. The lowest band is the template strand DNA22, which acted here as in internal negative control for binding. TFAM binding was performed with 0.2µM of DNA substrates and the indicated protein concentration in the same conditions shown in Figure 6.

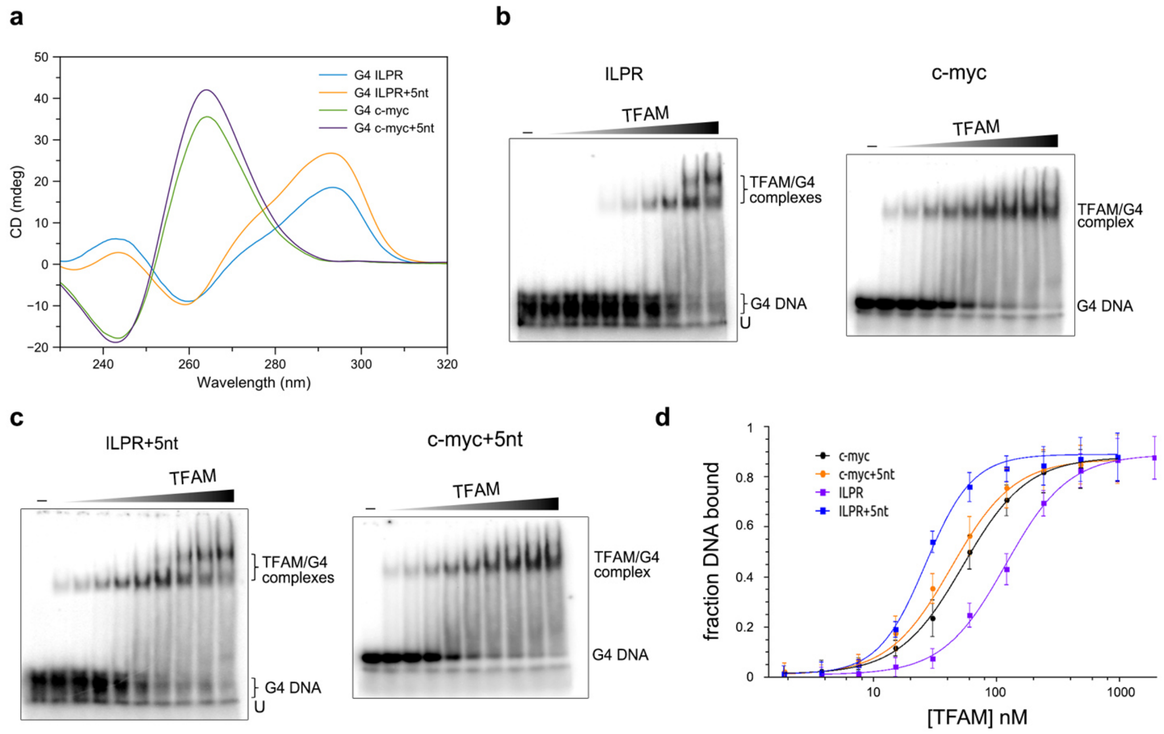

Supplementary Figure S4. TFAM binds to intramolecular G4-DNAs from c-myc and ILPR. (a) CD Spectra in 10mM sodium cacodylate (pH6.8) and 50mM KCl of the ILPR (blue), ILPR+5nt (orange), c-myc (green), c-myc+5nt (purple) after G4 folding in 100mM KCl. Sequences are shown in Table 1. **(b)** Band-shift assay with ^{32}P-labeled ILPR or ^{32}P-labeled c-myc (0.5nM) incubated with serial dilutions of TFAM (from 1.5nM to 0.5 µM). **(c)** Band-shift assay with ^{32}P-labeled-labeled ILPR+5nt (0.5nM) or ^{32}P-labeled c-myc+5nt incubated with serial dilutions of TFAM (from 0.9nM to 250 nM). **(d)** EMSA titration for TFAM binding to the four G4.

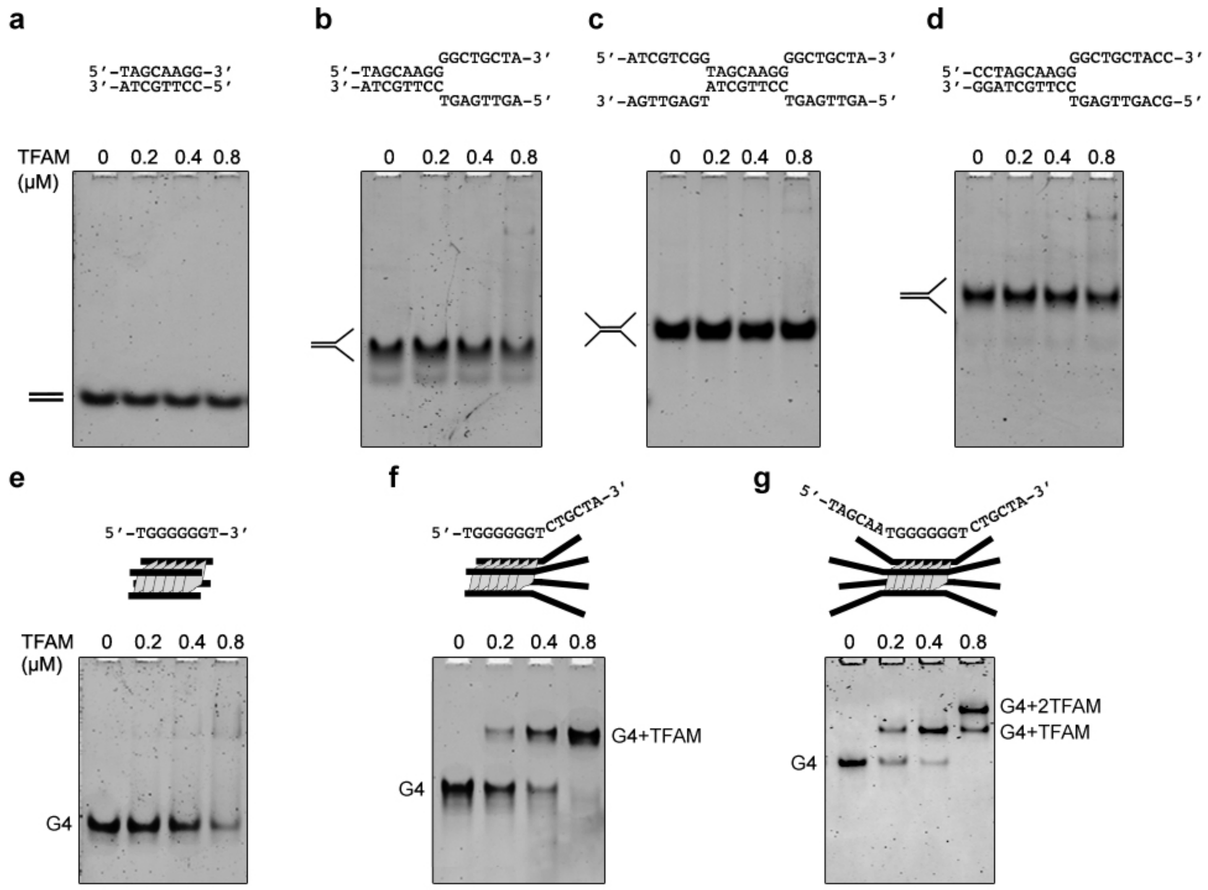

Supplementary Figure S5. TFAM binding to ds- and G4-DNA with single-stranded overhangs. The DNA sequences and the structures are indicated upon each gel. The gel and the binding buffer were complemented with 1mM $MgCl_2$ to stabilize the constructs. TFAM binding was performed with 0.2μM of DNA substrates and the indicated protein concentration in the same conditions than Figure 6.

Supplementary References

1. Wanrooij, P.H., Uhler, J.P., Shi, Y., Westerlund, F., Falkenberg, M. and Gustafsson, C.M. (2012) A hybrid G-quadruplex structure formed between RNA and DNA explains the extraordinary stability of the mitochondrial R-loop. *Nucleic Acids Res*, **40**, 10334-10344.
2. Chen, S.H., Suzuki, C.K. and Wu, S.H. (2008) Thermodynamic characterization of specific interactions between the human Lon protease and G-quartet DNA. *Nucleic Acids Res*, **36**, 1273-1287.
3. Hud, N. and Plavev, J. (2006) In Neidle, S. and Balasubramanian , S. (eds.), *Quadruplex Nucleic Acids*. RSC Publishing, Cambridge, pp. 100-129.
4. Mergny, J.L., Gros, J., De Cian, A., Bourdoncle, A., Rosu, F., Saccà, B., Guittat, L., Amrane, S., Mills, M., Alberti, P. *et al.* (2006) In Neidle, S. and Balasubramanian , S. (eds.), *Quadruplex Nucleic Acids*. RSC Publishing, Cambridge, pp. 31-80.
5. Rosu, F., Gabelica, V., Poncelet, H. and De Pauw, E. (2010) Tetramolecular G-quadruplex formation pathways studies by electrospray mass spectrometry. *Nucleic Acids Res*, **38**, 5217-5225.
6. Zheng, K.-W., Wu, R.-Y., He, Y.-D., Xiao, S., Zhang, J.-Y., Liu, J.-Q., Hao, Y.-H. and Tan, Z. (2014) A competitive formation of DNA:RNA hybrid G-quadruplex is responsible to the mitochondrial transcription termination at the DNA replication priming site. *Nucleic Acids Res*, **42**, 10832-10844.
